# Temporal Variations in Aboveground Biomass, Nutrient Content, and Ecological Stoichiometry in Young and Middle-Aged Stands of Chinese Fir Forests

**DOI:** 10.3390/plants13131877

**Published:** 2024-07-07

**Authors:** Zhiqiang Li, Can Mao, Qinxiang Wu, Yuanying Peng, Jun Wang, Bin Zhang, Sheng Zhang, Xiaocui Liang, Wende Yan, Xiaoyong Chen

**Affiliations:** 1 College of Life and Environmental Science, Central South University of Forestry and Technology, Changsha 410004, China; lizhiqiang@csuft.edu.cn (Z.L.);; 2 National Engineering Laboratory for Applied Forest Ecological Technology in Southern China, Changsha 410004, China; 3 Lutou National Station for Scientific Observation and Research of Forest Ecosystem in Hunan Province, Yueyang 410004, China; wqx0909@csuft.edu.cn (Q.W.);; 4 College of Forestry, Central South University of Forestry and Technology, Changsha 410004, China; 5 College of Arts and Sciences, Lewis University, Romeoville, IL 60446, USA; pengyu@lewisu.edu; 6 College of Arts and Sciences, Governors State University, University Park, IL 60484, USA

**Keywords:** Chinese fir forest, forest age, biomass, productivity, soil nutrients, ecological stoichiometry

## Abstract

Understanding the ecological dynamics of forest ecosystems, particularly the influence of forest age structure on soil carbon (C), nitrogen (N), and phosphorus (P) content, is crucial for effective forest management and conservation. This study aimed to investigate the nutrient storage and ecological stoichiometry across different-aged stands of Chinese fir forests. Soil samples were collected from various depths (0–15 cm, 15–30 cm, and 30–45 cm) across four age groups of Chinese fir forests (8-year-old, 12-year-old, 20-year-old, and 25-year-old) in the Forest Farm, Pingjiang County, China. Soil organic carbon (SOC), total nitrogen (TN), and total phosphorus (TP) were measured, and their stoichiometries were calculated. The results showed that both individual tree biomass and stand biomass, along with SOC, TN, and TP content, increased with stand age, highlighting the significant importance of stand age on biomass production and nutrient accumulation in forests. Specifically, soil C and P contents significantly increased as the forest aged, while variation in N content was relatively minor. Soil C/N and C/P ratios exhibited variation corresponding to forest age, suggesting alterations in the ecological stoichiometry characteristics of the forests over time. These findings are crucial for understanding the dynamics of ecosystem functioning and nutrient cycling within Chinese fir forests and provide a solid scientific basis for the effective management and conservation of these vital forest ecosystems.

## 1. Introduction

Forests are pivotal components of global nutrient cycles, climate regulation, biodiversity conservation, and ecosystem stability [1,2,3]. They act as significant carbon (C) sinks, sequestering atmospheric carbon dioxide through photosynthesis and storing it in biomass and soil organic matter [4]. Forests also play crucial roles in regulating nitrogen (N) and phosphorus (P) cycles, influencing nutrient availability and ecosystem productivity [5,6]. Moreover, forests provide essential habitats for diverse plant and animal species, contributing to biodiversity conservation and ecosystem resilience [7]. Understanding these intricate relationships is essential for sustainable land management and conservation efforts [6,8].

Forests structure, function, productivity and biodiversity vary with the forest stand ages. Young forests exhibit vigorous growth and high primary productivity, rapidly establishing canopy cover and biomass accumulation [9]. Middle-aged forests, spanning several decades to a century, represent stages of ecosystem maturation with stabilized growth and increasing canopy complexity [10], supporting rich biodiversity and serving as vital habitats [8]. In contrast, old-growth forests feature centuries-old trees and complex structures, representing ecosystem climax and stability [11], crucial for biodiversity maintenance, carbon storage, and ecosystem resilience [2]. Understanding forest dynamics across age classes is essential for effective management and conservation strategies. Previous studies have highlighted the importance of ecological stoichiometry, which examines the balance of elements such as carbon (C), nitrogen (N), and phosphorus (P) in ecosystems [12]. The stoichiometric ratios of these elements in plants and soils reflect fundamental ecological processes and can vary with factors such as nutrient availability, climate, and disturbance regimes [13]. Temporal variations in ecological stoichiometry and nutrient storage patterns in forest ecosystems are influenced by factors such as stand age, successional stage, and management practices [14]. Understanding the temporal variations in ecological stoichiometry and nutrient storage patterns within forest ecosystems is essential for effective management and conservation efforts and for implementing sustainable forest management strategies [10,15]. Ecological stoichiometry examines the relative proportions of chemical elements in ecological interactions, providing insights into nutrient dynamics and ecosystem functioning [12]. Temporal variations in stoichiometric ratios and nutrient storage distributions can result from seasonal changes, climatic fluctuations, and anthropogenic disturbances [16,17]. These variations have significant implications for forest productivity, nutrient cycling processes, and the resilience of forest ecosystems in the face of environmental changes [11].

Chinese fir (*Cunninghamia lanceolata*) forests are renowned for their rapid growth rates and economic significance in timber production [18]. It is one of the most economically and ecologically important tree species in China and is extensively cultivated in plantation forests across various regions due to its rapid growth, adaptability to diverse soil conditions, and high-quality timber production [6,19]. Chinese fir plantations cover millions of hectares in China and play significant roles in providing timber resources, stabilizing soil, and mitigating erosion [3]. Additionally, these plantations contribute to carbon sequestration and climate regulation, serving as carbon sinks in the landscape [20,21]. However, intensive management practices such as thinning, fertilization, and pest control in Chinese fir plantations can have both positive and negative impacts on ecosystem functions and biodiversity [22]. Concerns have also been raised about nutrient imbalances and ecosystem sustainability in these forests [14]. The dynamics of age structure in Chinese fir plantations and their interactions with soil and nutrients are poorly understood, posing a barrier to the development of sustainable forest management and conservation strategies in China.

This study aimed to investigate the temporal variations and interrelationships of soil nutrients in subtropical fir forests aged 8, 12, 20, and 25 years in the subtropical region of China. We hypothesized that (1) soil nutrient content in young fir forests would be lower than that in middle-aged fir forests, as younger forests may require more soil nutrients than they can generate, (2) nutrient content in surface soil would be higher than that in deep soil, with litter and biodiversity potentially influencing soil nutrient variations, and (3) patterns of carbon (C), nitrogen (N), and phosphorus (P) ecological stoichiometry would differ across the selected aged forests. The results of this study provide a scientific basis for further understanding nutrient dynamics in forest soil and for sustainable management of forest ecosystems.

## 2. Results

### 2.1. Aboveground Biomass of Chinese Fir Forests

Table 1 illustrates the progression of biomass accumulation and changes in stand characteristics across different age groups of Chinese fir forests. There were significant differences in aboveground biomass among the stand ages (*p* < 0.01). Younger forests, such as those aged 8 and 12 years, exhibit lower individual tree biomass but higher stand density compared to older stands. Specifically, the average individual biomass ranges from 17.3 kg/tree in 8-year-old stands to 34.5 kg/tree in 12-year-old stands. In contrast, older forests aged 20 and 25 years display significantly higher individual tree biomass, averaging 241.0 kg/tree and 237.9 kg/tree, respectively. These older stands also demonstrate higher total stand biomass, with 20-year-old stands averaging 102.3 t/ha and 25-year-old stands reaching 235.7 t/ha (Table 1).

The biomass accumulation increased with the age of the Chinese fir-dominated forests, evident at both individual tree and stand levels (Table 1). Forests at 8 years old exhibited relatively lower biomass, averaging 45.1 t/ha. As the forests matured, biomass significantly increased, reaching its peak at 25 years old with an average of 235.7 t/ha (Table 1). This trend underscores the substantial accumulation of biomass over time as the forests age.

### 2.2. The Contents of C, N, and P in Different Ages of Stands in Soil

**Figure 1:**
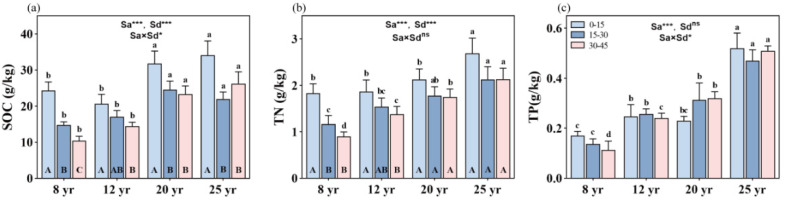
Variation in soil nutrient concentrations in the soil vertical profile across different stand ages of Chinese fir forests: (**a**) soil organic carbon (SOC), (**b**) soil total nitrogen (TN), and (**c**) total phosphorus (TP). Different lowercase letters indicate significant differences of soil nutrients at the same soil depth in different stand ages, while different uppercase letters indicate significant differences of soil nutrients in different soil depths at the same stand age. ** and *** denote *p* < 0.01 and *p* < 0.001, respectively, while “ns” indicates non-significance. “Sa” refers to stand age, and “Sd” refers to soil depth. Bars represent the mean ± standard error (n = 12).

Forest age significantly influences SOC, TN, and TP contents, with an observed trend of 25-year-old > 20-year-old > 12-year-old > 8-year-old stands (*p* < 0.01, Figure 1). Soil depth also exerts a significant effect on SOC and TN, resulting in a decrease in SOC and TN with increasing soil depth (*p* < 0.01, Figure 1a,b). However, TP does not exhibit a consistent trend with soil depth (Figure 1c). Moreover, the interactive effects of stand age and soil depth on SOC and TP were significant (*p* < 0.05, Figure 1a,c), but not on TN (Figure 1b), in the studied forests.

With stand aging, SOC content gradually increases. Specifically, SOC content increased by approximately 40%, 51%, and 144% at 0–15 cm, 15–30 cm, and 30–45 cm soil depths, respectively, in 25-year-old stands compared to 8-year-old stands. However, SOC content was significantly reduced from the topsoil (0–15 cm depth) to the mid-soil depth (15–30 cm depth) in all aged stands (*p* < 0.01, Figure 1a), with corresponding reductions of about 40%, 18%, 21%, and 36% of SOC between the two soil depths in the studied 12-, 20-, and 25-year-old stands, respectively. No significant differences in SOC content were found between the 15–30 cm and 30–45 cm soil depths in all aged stands, except in 8-year-old stands, where SOC content significantly decreased in soil depth from 15–30 cm to 30–45 cm.

On average, TN content gradually increases with increasing stand ages. Compared to 8-year-old stands, TN content increased by approximately 51%, 85%, and 138% at 0–15 cm, 15–30 cm, and 30–45 cm soil layers, respectively, in 25-year-old stands. Although soil TN contents were reduced from the topsoil to the deeper soil layers, significant differences in TN were found only in the 8- and 12-year-old stands, not in the 20- and 25-year-old stands (Figure 1b).

Stand age causes an increase in soil TP content in the studied forests, with approximately 215%, 238%, and 352% increases in TP at 0–15 cm, 15–30 cm, and 30–45 cm soil layers, respectively, in 25-year-old stands compared to 8-year-old stands (Figure 1c). With increasing soil depth, soil TP content was reduced in the 8- and 25-year-old stands but increased in the 12- and 20-year-old stands, respectively.

### 2.3. Stoichiometric Ratios of C, N, and P in Soil

**Figure 2:**
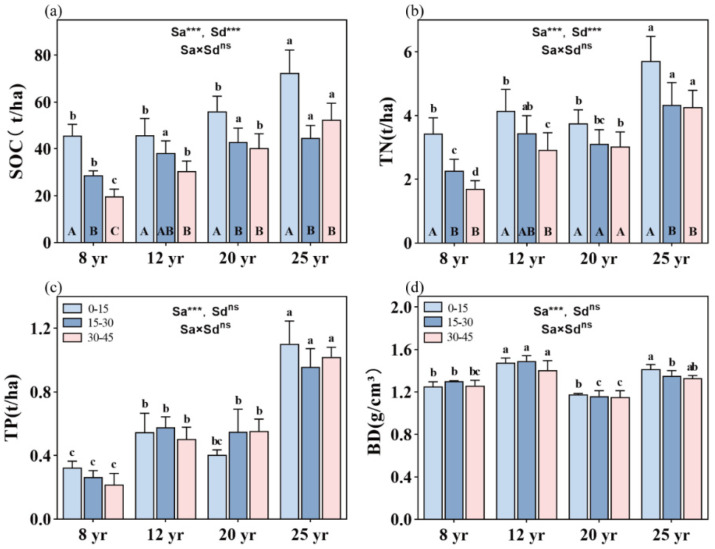
Distribution of SOC, TN, and TP storage and BD value in the soil vertical profile across different aged stands of Chinese fir forests: (**a**) SOC storage, (**b**) TN storage, (**c**) TP storage, and (**d**) BD value. Different lowercase letters indicate significant differences of soil nutrients at the same soil depth in different stand ages, while different uppercase letters indicate significant differences of soil nutrients in different soil depths at the same stand age. ** and *** denote *p* < 0.01 and *p* < 0.001, respectively, while “ns” indicates non-significance. “Sa” refers to stand age, and “Sd” refers to soil depth. Bars represent the mean ± standard error (n = 12).

Overall, stand age significantly influences SOC, TN, and TP storages, as well as soil BD in the studied forests (*p* < 0.01, Figure 2). SOC, TN, and TP storage are significantly higher in 25-year-old stands compared to the other three age categories (*p* < 0.01, Figure 2a–c). Soil depth also significantly affects SOC and TN storage in the studied forests, showing an overall decreasing trend with increasing stand age (*p* < 0.01, Figure 2). However, no significant difference in TP storage is found among the soil depths in the four age categories (Figure 2c). Furthermore, the interactive effects between stand age and soil layer do not significantly impact SOC, TN, and TP storage in the studied forests (Figure 2).

The total SOC storage increases with the aging of the forest, following a sequence of 8-year-old forests (93 t C/ha) < 12-year-old forests (113.5 t C/ha) < 20-year-old forests (137.5 t C/ha) < 25-year-old forests (167 t C/ha). SOC storage is notably higher in the topsoil compared to deeper soil layers. However, no significant difference in SOC storage is observed between the deeper soil layers in all studied stands, except in the 8-year-old stands, where SOC storage is significantly higher in the 15–30 cm soil layer than in the 30–45 cm soil layer (Figure 2a). Soil TN storage ranges from the minimum value found in the 8-year-old forests (7.5 t/ha) to the maximum value found in the 25-year-old forests (14.3 t/ha). With increasing soil depth, soil TN storage decreases, but a significant difference in TN storage between the 0–15 cm and 15–45 cm soil depths is observed in 8-, 12-, and 25-year-old stands.

Soil TP storage accumulates with stand aging in the following order: 8-year-old forests (0.9 t/ha) < 12-year-old forests (1.6 t/ha) ≈ 20-year-old forests (1.6 t/ha) < 25-year-old forests (3.3 t/ha) (Figure 2c). Soil bulk density (BD) is significantly higher in the 12-year-old stands (1.443 g/cm^3^) and 25-year-old stands (1.343 g/cm^3^) than in the 8-year-old stands (1.260 g/cm^3^) and 20-year-old plots (1.170 g/cm^3^) (*p* < 0.01, Figure 2d).

### 2.4. The Overall Average Contents of C, N, and P in the Entire Sample Plot

**Figure 3:**
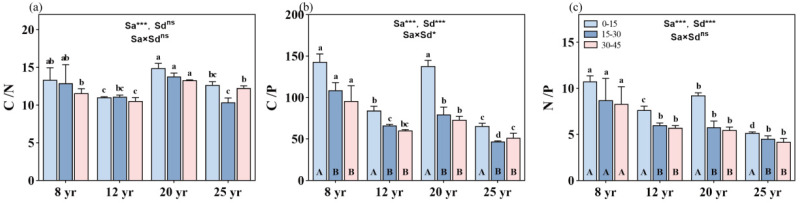
Changes in C, N, and P stoichiometry across different soil depths in different aged stands of Chinese fir forests: (**a**) C/N ratio, (**b**) C/P ratio, and (**c**) N/P ratio. Different lowercase letters indicate significant differences of soil nutrients at the same soil depth in different stand ages, while different uppercase letters indicate significant differences of soil nutrients in different soil depths at the same stand age. ** and *** denote *p* < 0.01 and *p* < 0.001, respectively, while “ns” indicates non-significance. “Sa” refers to stand age, and “Sd” refers to soil depth. Bars represent the mean ± standard error (n = 12).

The age of the forests significantly influenced the C/N, C/P, and N/P ratios (*p* < 0.01, Figure 3). Both the C/P and N/P ratios exhibited an inverse decreasing trend with increasing stand age (*p* < 0.01, Figure 3). Additionally, different soil layers did not show a significant change in C/N and N/P with stand age (Figure 3a,c), while the interaction between stand age and soil layer significantly affected soil C/P (*p* < 0.05, Figure 3b).

The soil C/N ratio decreased with increasing soil depth, but there were no significant differences in the C/N ratio among various soil depths within the same-aged stands (Figure 3a). On average, the C/N ratio was approximately 12.5, 10.8, 14, and 11.5 in the soil profile (0–45 cm depth) in the 8-, 12-, 20-, and 25-year-old stands, respectively. The stoichiometric characteristics of the soil C/P ratio at different soil layers in the 8- and 20-year-old stands were significantly higher than that at the corresponding soil layers in the other two aged stands (*p* < 0.01, Figure 3b). The soil C/P ratio decreased with increasing soil depth in all studied forests, but the significant difference in the soil C/P ratio was only found from the 0–15 cm soil layer to the 15–30 cm soil layer. No significant difference in the soil C/P ratio was found between the 15–30 cm and 30–45 cm soil layers in the aged forests. On average, the C/P ratio was approximately 144, 87, 140, and 64 in the topsoil layer, and 98, 64, 74, and 49 in the deeper soil layers (15–45 cm depth) in the 8-, 12-, 20-, and 25-year-old stands, respectively.

In general, the soil N/P ratio decreased with the increase in stand ages (Figure 3c), with mean values of the N/P ratio of 9.0, 6.6, 6.7, and 4.7 in 8-, 12-, 20-, and 25-year-old stands, respectively. The soil N/P ratio significantly decreased from the 0–15 cm layer to the 15–30 cm layer in all aged stands of Chinese fir forests in the study site, except in the 8-year-old stands, where no significant difference in the N/P ratio was found among the different soil depths. At the deeper soil layers (15–45 cm soil depth), there were no significant differences in the N/P ratio among the 12-, 20-, and 25-year-old stands.

## 3. Materials and Methods

### 3.1. Study Site

**Figure 4:**
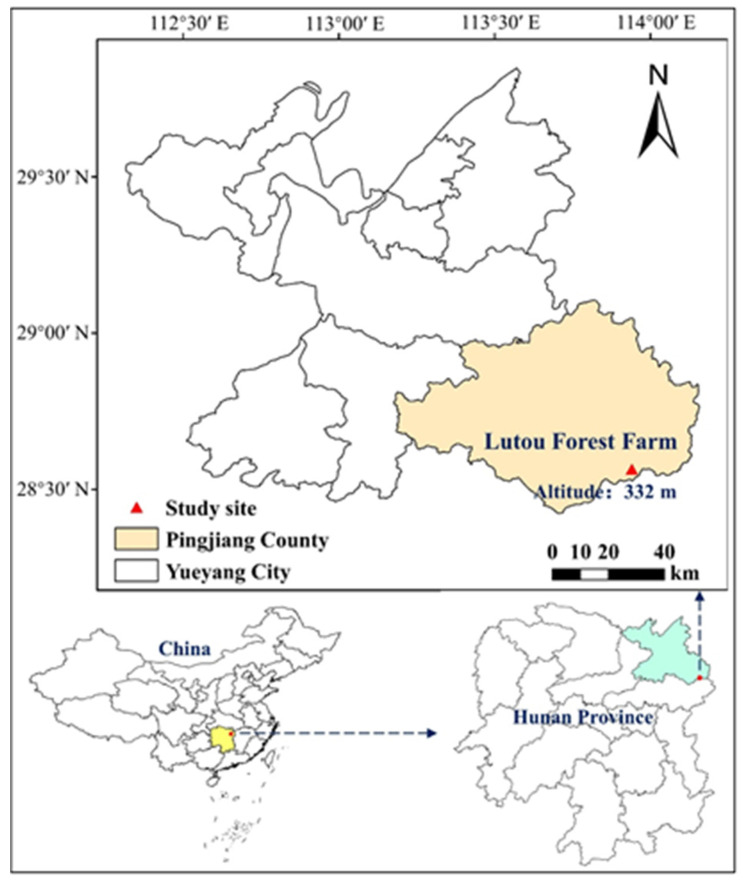
The study site is located at Lutou Forest Farm, Pingjiang County, Hunan Province, China.

This study was conducted in the Lutou Forest Farm, Pingjiang County, Hunan Province, China (E113°51′52″–113°58′24″, N28°31′17″–28°38′00″) (Figure 4). The region exhibits an elevation range from 124 m to 1272.5 m and an average slope of approximately 30 degrees. Situated in the subtropical to temperate transition zone of central China, the forest farm experiences a humid continental climate. The average annual temperature is 16.8 °C, with around 1360 h of annual sunshine, an average air humidity of 82%, and an average annual precipitation of 1968.8 mm. The vegetation in the forest farm zone primarily comprises evergreen broad-leaved forests featuring diverse stand types. For this study, Chinese fir forests of four age categories were selected, including 8 years, 12 years, 20 years, and 25 years.

The soil in our study sites, classified as mountain yellow-brown (Ellusols) forest soil, originated from a long-standing forest ecosystem that was primarily composed of mixed deciduous and coniferous trees. These old-growth forests were selectively logged, and subsequently, Chinese fir (*Cunninghamia lanceolata* Lamb. Hook) forests were planted. The genesis of this soil profile is deeply rooted in the complex interaction of organic matter from the previous forest, climatic conditions, and the underlying parent material, primarily weathered granite. The dominant tree species in these fir communities are fir trees, alongside associated tree species such as Chinese sweet gum (*Liquidambar formosana* Hance)*,* Masson’s pine (*Pinus massoniana* Lamb.), Chinese catalpa (*Catalpa ovata* G. Don), Red maple (*Acer rubrum* L.), and Chinese alder (*Alnus trabeculosa* Hand.-Mazz.). The understory shrubs consist of *Maesa japonica* (Thunb.) Moritzi ex Zoll., *Smilax china* L., *Ficus benjamina* L., and *Vernicia fordii* (Hemsl.) Airy Shaw, while the herbs include *Woodwardia japonica* (L.f.) Sm., *Lophatherum gracile* Brongn., *Lygodium japonicum* (Thunb.) Sw., and *Parathelypteris glanduligera* (Kunze) Ching in the forests.

### 3.2. Experimental Design and Soil Sampling

This study was conducted in 2022. The study sites consisted of four age groups of Chinese fir plantations with similar site conditions: 8 years old, 12 years old, 20 years old, and 25 years old. These plantations were established in 1997, 2002, 2010, and 2014, respectively. The four stand ages of Chinese fir plantations were selected in the study area under similar site conditions, apart from their distinct age groups. All other factors, such as slope degree, position, and direction, were kept consistent.

The study employed a split-plot design, with different stand ages (8 years, 12 years, 20 years, 25 years) as the main factor and soil depth (0–15, 15–30, 30–45 cm) as the subplot factor. Three replicate plots, each measuring 30 m × 30 m, were established at the study site. Information on the stand characteristics for each age category is provided in Table 1. Within each plot, diagonal sampling was conducted at soil depths of 0–15, 15–30, and 30–45 cm, respectively. A total of 36 soil samples (4 stand ages × 3 soil depths × 3 replicates) were collected and transported to the laboratory in iceboxes. Upon arrival, the samples underwent sieving through a 2 mm mesh to eliminate roots, woody debris, and stones. The sieved soil samples were air-dried and passed through a 0.149 mm sieve for the determination of soil organic carbon (SOC), total nitrogen (TN), and total phosphorus (TP).

### 3.3. Measurements of Biomass in Chinese Fir Forests

This study measured the biomass of 8-, 12-, 20-, and 25-year-old Chinese fir forests. Tree biomass was determined using the harvest method as described by Wang et al. (2023) [20]. Briefly, the diameter at breast height (DBH, measured at 1.3 m above ground level) and the height of all trees were recorded in each plot. Six sample trees, representing a range of DBH and heights within each plot, were selected for detailed measurements. Once a sample tree was felled, its main stem was cut into two sections and weighed. A stem disc approximately 2 cm wide was cut from each end of these sections to determine the weight of bark and wood, as well as the ratio of fresh to dry weight for both components. This ratio was then used to calculate the total dry weight of the tree.

Simultaneously, all branches with leaves were removed in 2 m vertical layers from the bottom to the top of the crown. Leaves were stripped from the branches, and the fresh weights of both branches and leaves at each layer were recorded. The branch-to-leaf weight ratio was calculated. Subsamples of branches and leaves were taken for dry weight determination.

The roots of each sampled tree were carefully excavated from different soil layers (0–15 cm, 15–30 cm, and 30–45 cm depths), and their fresh weights were determined. Subsamples of the roots were also collected for dry weight determination.

Subsamples were oven-dried at 70 °C until they reached a constant weight. The biomass and components of biomass for each sampled tree were then calculated. The dry weight data obtained from the sample trees were used to develop allometric regression equations through stepwise regression with tree diameter and height as predictors. These equations were used to estimate the biomass of Chinese fir stands at different ages.

### 3.4. Soil Sampling Analysis

The soil bulk density (SBD) was determined by drying fresh soil samples at 105 °C until a constant weight was achieved. Soil organic carbon (SOC) concentrations were measured using the K_2_Cr_2_O_7_-H_2_SO_4_ oxidation method, which quantifies the amount of carbon present in organic compounds within the soil. Total nitrogen (TN) content was determined using the micro-Kjeldahl method combined with steam distillation, a widely used technique for nitrogen analysis that involves digestion and distillation of soil samples to release and quantify nitrogen compounds. Total phosphorus (TP) levels were measured using the molybdenum-antimony colorimetric method, which involves the reaction of phosphorus with reagents to form a colored complex that can be quantified spectrophotometrically [15]. These analytical methods were employed to assess the physicochemical properties of soils collected from different stand ages, as outlined in Table 1.

### 3.5. Statistical Data Analysis

The statistical analysis conducted in this study involved a two-way ANOVA to examine the effects of stand ages and soil depths on soil nutrient variables, including soil SOC, TN, and TP. The study employed a split-plot design, treating stand ages as the main factor and soil depths as the subplot factor. Three replicate plots were established for each combination of stand age and soil depth, resulting in a total of 36 soil samples collected from the study site. Upon sample collection, soil samples underwent preparation procedures, including sieving, and air drying, before the determination of SOC, TN, and TP concentrations. The ANOVA analysis assessed the main effects of stand ages and soil depths, as well as their interaction effects, on soil nutrient variables. Post hoc tests, such as Tukey’s HSD, were performed to identify specific differences between stand ages and soil depths if significant interaction effects were detected. SAS v9.4 software (SAS Institute, Inc, Cary, NC, USA 2019)was utilized for data analysis in the study.

## 4. Discussion

### 4.1. Biomass Accumulation with Changes in Stand Ages

In this study, we observed a consistent increase in both individual tree and stand biomass with the age of Chinese fir forests, following the sequence: 8-year-old forests < 12-year-old forests < 20-year-old forests < 25-year-old forests. This finding is consistent with previous research emphasizing the influence of stand age on biomass accumulation in forest ecosystems [3,23]. At 8 years old, the relatively lower biomass levels observed in the forest stands could be attributed to the early stage of stand development and the smaller size of individual trees. This stage is characterized by the establishment of the forest canopy with relatively rapid tree height growth, resulting in limited biomass accumulation [24]. As the forests mature, biomass accumulation increases significantly. By 12 years of age, the biomass nearly doubled, indicating a period of rapid growth and canopy development. This result aligns with studies demonstrating exponential biomass accumulation during the mid-successional stages of forest development [25]. By 20 years of age, the forests showed a substantial increase in biomass, reaching an average of 102.3 tons per hectare. This phase represents a critical period in forest development where biomass accumulation is stabilized, reflecting the attainment of a nearly mature forest structure characterized by a closed canopy and maximum tree size [26]. Finally, at 25 years old, the forests reached their peak biomass levels, with an average of 235.65 tons per hectare. Our study underscores the importance of aging stands in contributing to biomass storage and carbon sequestration, highlighting the role of mature forests in mitigating climate change and providing essential ecosystem services [27].

### 4.2. Variation of Soil Nutrient Content with Changes in Stand Age

Our study found that mature forest stands exhibited markedly higher levels of SOC, TN, and TP contents compared to younger stands (see Figure 1). The SOC, TN, and TP storage were markedly higher in 25-year-old stands compared to younger stands in this study (Figure 1), indicating a progressive accumulation of organic matter and nutrients over time [28]. This accumulation was likely driven by increased litterfall, root turnover, and organic debris input in mature stands, fostering soil nutrient enrichment. This accumulation could be attributed to various factors, including the gradual decomposition of plant litter, biomass accumulation, and increased root turnover in older forests [28,29].

The observed trend of increasing soil nutrient content with increasing forest stand age aligns with findings from previous experiments, highlighting the progressive accumulation of soil organic matter and nutrients over time in forest ecosystems [6,9]. This accumulation is attributed to factors such as litter decomposition, root turnover, and nutrient cycling processes, which become more pronounced in older forests due to greater biomass and organic matter inputs [10,27]. Previous studies have also found that young stands of Chinese fir typically exhibit higher nutrient uptake rates and nutrient cycling dynamics as they establish vigorous root systems and canopy development [30]. These processes are critical for nutrient redistribution within the soil profile and contribute to ecosystem stability and resilience against environmental stressors [14]. In middle-aged stands, nutrient cycling becomes more regulated, with soil nutrient pools supporting sustained growth and productivity while contributing to long-term soil fertility and ecosystem functioning [31].

### 4.3. Soil Nutrient Content with Changes in Soil Depth in Chinese Fir Stands

Across all forest ages, there was a consistent decline in SOC and TN concentrations with increasing soil depth, indicating higher nutrient levels in surface layers compared to deeper layers. This pattern reflects the predominant inputs of organic matter near the soil surface from litterfall and root activity, leading to enhanced nutrient accumulation in the topsoil [32,33]. However, TP did not exhibit a consistent trend with soil depth, suggesting a more intricate interplay between P dynamics and soil properties. Additionally, interactive effects between stand age and soil depth on SOC and TP contents were found in this study, indicating the complex nature of forest–soil interactions and underscoring the need to consider both temporal and spatial dynamics in sustainable forest ecosystem management [34]. Understanding the nuanced relationships between forest development and soil processes is essential for accurately predicting soil nutrient dynamics and devising effective conservation and management strategies.

The significant decrease in SOC and TN with increasing soil depth is consistent with findings from other studies, indicating the influence of soil profile characteristics on nutrient distribution [30,35]. Soil organic matter and N are primarily concentrated in the surface soil layers due to organic inputs from aboveground litterfall and root exudates, as well as microbial activity [11]. In contrast, TP shows no significant variation with soil depth, suggesting different patterns of P distribution and mobility compared to C and N in forest soils [36]. The interaction between stand age and soil depth further influences soil nutrient dynamics, particularly for SOC and TP. This interaction effect likely results from variations in litter quality, root distribution, and microbial activity across different stand ages and soil depths, which influence nutrient cycling processes and organic matter decomposition rates [2,36]. While TN does not exhibit significant changes across soil layers with stand age, the combined effects of stand age and soil depth on soil N dynamics warrant further investigation to elucidate underlying mechanisms [11].

Soil depth played a crucial role in modulating soil nutrient distribution, with implications for ecosystem functioning. An overall decreasing trend was observed in SOC and TN storage with increasing soil depth in the studied forests, underscoring the importance of surface soil layers in nutrient retention [33]. However, no significant difference in TP storage was observed among different soil depths, suggesting a relatively uniform distribution of P within the soil profile. Interestingly, the interactive effects between stand age and soil layer did not significantly influence SOC, TN, and TP storage, suggesting the robustness of the observed trends irrespective of soil depth variations [37].

### 4.4. Soil Nutrient Stoichiometry of C/N, C/P, and N/P with Changes in Stand Ages of Chinese Fir Stands

The age of the forest stands significantly influenced the C/N, C/P, and N/P ratios in the study sites (Figure 3), reflecting variations in soil nutrient stoichiometry across different stages of forest development. The results were consistent with the findings of previous studies, demonstrating the dynamic nature of soil nutrient ratios in response to changes in forest stand age and ecosystem processes [23,38]. The observed decreasing trends in C/P and N/P ratios with increasing stand age indicated potential shifts in nutrient cycling dynamics, with mature forests likely exhibiting greater nutrient retention and recycling capacities compared to younger stands [14,27]. The significant differences in soil C/P ratios between the 8-year-old and 20-year-old stands compared to other stand ages suggested variations in nutrient allocation and availability across different stages of forest succession. These differences might be attributed to factors such as litter quality, root biomass distribution, and microbial activity, which influenced the decomposition and mineralization of organic matter and subsequent nutrient release [3,15].

### 4.5. Soil Nutrient Stoichiometry with Changes in Soil Depth and Their Interaction with Stand Ages in Chinese Fir Stands

Soil depth played a crucial role in shaping soil nutrient stoichiometry, with deeper soil layers exhibiting lower C/P and N/P ratios compared to surface layers. This pattern reflected the vertical redistribution of nutrients within the soil profile, driven by processes such as leaching, root uptake, and microbial decomposition [3,36]. The interaction between stand age and soil layer further modulates soil nutrient ratios, particularly for C/P, highlighting the complex interplay between forest development and soil nutrient dynamics. This interaction effect likely resulted from the combined influences of litter inputs, root turnover, and microbial activity across different stand ages and soil depths, influencing nutrient cycling processes and stoichiometric relationships [15,21]. While soil C/N and N/P ratios did not show significant changes with stand age across different soil layers, the observed variations in C/P ratios underscored the importance of considering both horizontal and vertical dimensions of soil nutrient distribution in forest ecosystems.

However, the magnitude of soil nutrient accumulation and the rates of change in soil properties might vary depending on factors such as climate, soil type, and management practices. For example, studies conducted in temperate forests have reported faster rates of soil organic matter accumulation compared to subtropical or tropical forests due to differences in decomposition rates and litter quality [10,32]. Additionally, forest management activities such as thinning, fertilization, and afforestation could influence soil nutrient cycling processes and alter soil nutrient stoichiometry over relatively short time scales [15].

Comparative analyses across different forest ecosystems could provide valuable insights into the mechanisms driving soil nutrient dynamics and the resilience of forest ecosystems to environmental changes. For example, studies comparing soil nutrient stoichiometry in natural forests versus managed plantations could elucidate the long-term impacts of human interventions on soil fertility and ecosystem functioning [39,40]. Additionally, cross-site comparisons could help identify common patterns and drivers of soil nutrient dynamics across diverse climatic and biogeographic gradients, contributing to a more comprehensive understanding of global soil C and nutrient cycling processes [14].

## 5. Conclusions

Our study highlights the significant influence of forest stand age and soil depth on soil nutrient dynamics and ecological stoichiometric characteristics in Chinese fir forests. As forests age, there is a notable increase in biomass accumulation and nutrient content, particularly soil organic carbon, total nitrogen, and total phosphorus. This trend highlights the role of forest maturation in enhancing nutrient stocks and ecosystem productivity.

With increasing stand age, we observed a marked increase in individual tree and total stand biomass, indicating a shift from high-density young stands to more biomass-intensive older stands. Similarly, soil nutrient contents, such as SOC, TN, and TP, showed substantial increases with stand age, particularly at deeper soil levels, reflecting enhanced nutrient accumulation and stratification as the forest matures.

The interaction between stand age and soil depth revealed complex nutrient dynamics. While SOC and TN levels decreased with soil depth, the patterns for TP varied, indicating diverse factors influencing nutrient distribution. Additionally, changes in stoichiometric ratios of C/N, C/P, and N/P with stand age and soil depth suggest significant alterations in nutrient availability and cycling processes, crucial for understanding long-term forest ecosystem health and resilience.

These findings provide valuable insights into the nutrient dynamics of Chinese fir forests, emphasizing the importance of both stand age and soil depth in managing nutrient accumulation and distribution. Such knowledge is vital for developing sustainable forest management practices that enhance ecosystem health. Future research should assess deeper the mechanisms driving these nutrient patterns and their implications for long-term forest ecosystem functioning and resilience.

## Figures and Tables

**Table 1:** Characteristics of the selected four aged stands of Chinese fir forests in the study site *.

Stand Age (Year)	Stand Density (Tree/ha)	DBH (cm)	Tree Height (m)	Individual Biomass (kg/Tree)	Stand Biomass (t/ha)
8	2604	9.2	6.5	17.3	45.1
12	2745	11.5	9.8	34.5	94.8
20	425	24.7	16.5	241.0	102.3
25	991	25.5	16.6	237.9	235.7

* The tree species composition for 8-year-old stands comprised 94.6% Chinese fir, 2.7% Chinese sour plum, 1.6% Masson pine, and 1.1% broadleaf species. For 12-year-old stands, the tree species composition consisted of 90.2% Chinese fir, 5.2% Eucalyptus, 2.6% Chinese sour plum, and 2.1% broadleaf species. In 20-year-old stands, the tree species composition was 90% Chinese fir, 7% Eucalyptus, and 3% maple-leaf ash. Finally, for 25-year-old stands, the tree species composition included 90% Chinese fir, 4.2% Schima superba, and 5.7% broadleaf species.

## Data Availability

The data presented in this study are available upon request from the corresponding author.
